# The effects of external cues on cross-border e-commerce product sales: An application of the elaboration likelihood model

**DOI:** 10.1371/journal.pone.0293462

**Published:** 2023-11-09

**Authors:** Meiyu Fang, Ziling Deng, Junhui Guo

**Affiliations:** School of Economics and Management, Zhejiang University of Science and Technology, Hangzhou, China; UTM Skudai: Universiti Teknologi Malaysia, MALAYSIA

## Abstract

In response to the soaring demand for imported goods among Chinese consumers, this study innovatively investigates the role of external cues on consumer behavior on cross-border e-commerce platforms, utilizing advanced data crawling techniques to extract data from Tmall Global. Guided by the Elaboration Likelihood Model, this research unveils key determinants affecting consumer purchasing decisions, contributing novel insights to e-commerce literature and methodologies. Our analysis discovers that increased picture comments significantly boost sales and that source credibility, product collections, and price discounts also play pivotal roles, especially for experiential products. We further explore a nuanced, inverted U-shaped relationship between product title length and sales, offering a foundational understanding of non-linear relationships in consumer behavior and presenting practical implications for enhancing marketing strategies. This study, while acknowledging limitations like data access constraints, provides strategic insights into optimizing product information presentation and broadens understanding of consumer decision-making processes, thus adding substantial value to ongoing e-commerce discourse.

## Introduction

In March 2020, the National Development and Reform Commission (NDRC) and other governmental entities issued the "Implementation Opinions." This directive underscored the need to align with evolving consumption trends, strengthen the overall consumption ecosystem, and bolster the domestic market [1]. The evolving landscape of China’s consumer demand increasingly favors diversity and enriched experiences over sheer quantity. In response to this paradigm shift, the proactive development of imported cross-border e-commerce strategically allocates resources to cater to the evolving preferences of consumers, addressing the burgeoning demand for imported goods within the framework of the new dual circulation development pattern.

The evolution of China’s imported cross-border e-commerce sector has ushered in a transformative shopping experience for consumers, granting them access to an abundant array of imported products from the comfort of their homes. Nevertheless, this distinctive online shopping format has exacerbated information asymmetry between buyers and sellers. Unlike domestic e-commerce platforms, consumers navigating imported cross-border e-commerce platforms are confronted with multifaceted considerations, including geographical distance, tariff implications, and return policies. These complexities magnify the inherent uncertainties associated with online shopping, often culminating in heightened perceived risks—a concept expounded upon by Bauer [2].

Extensive research has affirmed that perceived risk is a significant deterrent to online purchases. Complete product information and satisfactory service provision act as potent factors in curtailing perceived risk, exerting a positive influence on purchase intentions [3]. Scholars have also posited that augmented information quality, reliability, and after-sale support on websites not only streamline consumer purchase endeavors but also diminish perceived risk, ultimately enhancing website efficacy [4]. According to cue utilization theory, when virtual network judgments elude consumers, reliance on external cues becomes pivotal in shaping product assessments. Employing external cues can effectively mitigate perceived risk, thereby reducing the uncertainty tied to online shopping and facilitating purchase decisions [5].

Moreover, extant research has validated the substantial influence of experience-based and search-based products on the relationship between various external cues and sales within domestic e-commerce platforms, the specific emphasis on product type in the domain of imported cross-border e-commerce remains relatively underdeveloped [6]. Given the multifaceted nature of website information and the intricate external cues inherent in imported cross-border e-commerce, variations in consumer focus and information processing pathways across different products types are plausible. Consequently, an examination of the moderating role of product types can yield deeper insights into the interplay between external cues and sales in the context of imported cross-border e-commerce.

Furthermore, discussing the pronounced information asymmetry between buyers and sellers in the e-commerce environment is of paramount importance. Online comments have emerged as a critical source of information pertaining to the quality of goods, significantly mitigating consumers’ perceived risks during the online purchasing process. These online comments wield substantial influence over consumers’ purchasing decisions, consequently exerting a pronounced impact on the sales of products. While recent research on cross-border e-commerce products sales has predominantly focused on standard online comment indicators such as the number of comments, comment characteristics, and positive feedback rates [7, 8], it has oftentimes overlooked other pivotal factors unique to cross-border e-commerce platforms [9].

In real cross-border shopping environments, consumers not only depend on standard online comment indicators but also take into account other factors, including the source of product information, price discounts, and the number of collections. Thus, this paper expands the purview of research variables by incorporating new indicators such as the quality of imported product information and the credibility of product sources. This expansion serves to enrich the current repertoire of research variables used in the examination of product sales on cross-border e-commerce platforms.

Moreover, in the sphere of research models, scholars have predominantly embraced traditional information system models such as the information system success model and the technology acceptance model. Few researchers have ventured into the elaboration likelihood model (ELM). This paper employs the ELM model to systematically categorize external cues on cross-border e-commerce platforms into two dimensions: the central path and the peripheral path. A meticulous analysis of these paths’ influence on product sales offers a more lucid comprehension of the information processing mechanisms underpinning consumers’ online purchasing decisions. In doing so, it enriches the repertoire of research models within the field of e-commerce consumer behavior.

In addition to investigating linear relationships, this paper delves into non-linear relationships, particularly the nuanced relationship between the length of products’ titles and their sales. This research broadens the horizons of our understanding regarding the impact of product sales and lays the groundwork for future studies exploring non-linear relationships between other external cues and product sales in e-commerce.

The paper is structured as follows: The Literature Review provides a comprehensive overview and establishes the theoretical foundations of the study. The Research Hypothesis and Model section outlines the research hypotheses and presents the conceptual framework. In the Materials and Methods section, we detail the data sources, variable selection criteria, and provide descriptive statistics. The Results section conducts empirical analysis and reports the findings. The Discussion section engages in a critical evaluation of theoretical contributions, limitations, and future research directions. Finally, the Conclusions and Recommendations section offers conclusive insights and policy implications derived from the research.

## Literature review

In this review, we present a framework addressing the role of external cues in online consumer purchasing decisions. Through analysis of established and emerging studies, we identify a consistent influence of these cues on consumer choices in e-commerce settings. This investigation integrates traditional consumer behavior paradigms with the nuances of digital purchasing, underscoring the significance of external cues.

### Current research on external cues and consumers’ online purchase behavior

Cue theory, which examines the influence of signals on people’s perceptions, judgments, and decision-making processes, is relevant in the context of e-commerce. Cox introduced Cue Utilization Theory, proposing that product information can be distilled into cues such as price, brand, color, and origin. Consumers employ these cues to evaluate product quality during the purchasing process [10]. Olson expanded on this theory by categorizing cues as internal or external. Internal cues are inherent product characteristics that are difficult to change, while external cues encompass features subject to external control, such as brand, price, and origin [11]. Cue Utilization Theory suggests that these cues serve as indicators for consumers to assess product quality. Given the virtual nature of online shopping environments, internal cues are often elusive, causing consumers to increasingly rely on external cues to judge product quality, making them influential factors in consumers’ online purchasing behavior in e-commerce scenarios.

Wang and Qu classified external cues in online buying scenarios into five major types: value perception cues, perceptual situational cues, quality and safety signals, information-driven cues, and identity reliance cues. Their study, adopting a customer-centric approach, delved into the relationship between situational cues and consumers’ online shopping intentions [12]. Cai and Zhang examined the impact of cross-border e-commerce shopping platforms on consumers’ online purchase behavior. They identified availability, product price, sales, online reviews, and after-sales service as the external cues of cross-border e-commerce platforms and concluded that after-sales service exerts the greatest influence on consumers’ cross-border online purchase behavior [13]. Mao focused on three geographical cues specific to cross-border e-commerce, namely brand country, production country, and shipping country, and found significant effects of all three cues on consumers’ cross-border online shopping behavior [14].

Existing studies on the relationship between external cues in cross-border e-commerce and consumers’ online purchasing behavior primarily adopt a consumer-centric perspective, employing questionnaires to examine consumers’ perceived behavior at the website level. However, objective data from web pages have not been extensively explored. Assessing consumers’ online purchasing behavior through product sales, a direct indicator of their actual shopping preferences, offers a more comprehensive understanding of their decision-making processes. Hence, this study aims to utilize objective sales data from cross-border e-commerce platforms to investigate how product exterior signals influence customers’ online purchasing behavior.

### Current research on ELM and consumers’ online purchase behavior

The Elaboration Likelihood Model (ELM) is a significant theoretical model in information processing research, proposed by Petty and Cacioppo in 1986. It posits that the persuasion process involves two paths: the central path and the peripheral path. The central path involves in-depth consideration of information quality, leading to more persistent attitudes. In contrast, the peripheral path relies on simpler judgments based on source credibility and situational factors [15].

Scholars have recently applied the ELM model to examine online shopping behavior. For instance, Zhu and Yuan investigated the impact of online review quality and reputation on consumers’ purchase intentions, finding that product involvement moderates this relationship [16]. Lan Peng and Shi Li studied the influence of word-of-mouth quantity and quality on consumers’ purchase intentions using the participation theory and ELM model, with word-of-mouth quantity as the peripheral path and word-of-mouth quality as the central path [17]. Li Qi explored the effects of peripheral cues and central cues in live e-commerce, finding that peripheral cues and live information quality significantly impact users’ perceptions of information utility [18].

Based on these studies, information quality is typically considered the central path, while source credibility is viewed as the peripheral path in the research model. The difference lies in the time and effort required for information processing. Given the abundance of information available for imported goods, consumers evaluate the quality by assessing its relevance, vividness, and sufficiency. This central path involves substantial time and effort. On the other hand, consumers can judge the credibility of information sources for imported goods based on the type of stores displayed on the product page, relying on shallow thinking as a peripheral path. Therefore, this study on cross-border e-commerce consumer behavior encompasses the applicability of information quality and source reliability for imported goods.

### Current research on perceived risk

Scholars have extensively examined decision-makers’ risk attitudes. Brown et al. conducted an empirical study revealing that decision-makers’ risk aversion profoundly influences strategy choices [19]. In cross-border e-commerce consumer behavior research, Xie Wenlei posited that consumers weigh high purchase risks against the allure of items to mitigate risk aversion concerns [20]. Wellson underscored the distinct risks posed by online shopping due to the Internet’s unique nature and formulated a dynamic model linking perceived risk and online purchase willingness [21]. Nena consolidated perceived online shopping risks into categories like economic, quality, social, psychological, and time-related [22]. Alok categorized consumers as risk-neutral or risk-averse, constructing a decision-making model incorporating commodity search cost, price, and perceived risk [23]. Pappas et al. emphasized perceived risk’s significance in probing factors influencing consumers’ online buying behavior within international e-commerce [24]. The aforementioned studies have dissected consumers’ perceived risks in the shopping journey from diverse viewpoints. In the context of cross-border e-commerce platforms, trust between consumers and the platform is cultivated when external cues enhance perceived security, ultimately mitigating perceived risks. This reduction in perceived risk prompts positive purchasing behaviors among consumers.

### Current research on moderating effects of product types

Nelson’s classification of goods into search-based and experience-based categories is rooted in consumers’ varying information acquisition capabilities during e-commerce transactions [25]. Search-based goods are those that consumers can readily assess for quality prior to purchase, such as computers, watches, and household appliances. In contrast, experience-based goods, like toiletries and cosmetics, involve quality determination that necessitates personal interaction. Nelson highlights that consumer behaviors for search-based goods involve extensive information search due to their objective evaluability, while for experience-based goods, such behaviors are relatively subdued, given their subjectivity.

Current research underscores the influence of product type on consumers’ online purchasing behavior. Xiao et al. reveal that for search-based goods, consumers prioritize detailed product information, while for experiential goods, they tend to consider peer behaviors [26]. Jimenez et al. demonstrate that consumers gauge the quality of online purchases based on reviewers’ emotional attitudes towards the goods [27]. Li and Wu’s work indicates a more pronounced herd effect for experiential goods compared to search-based goods in online consumer contexts [28]. Notably, despite utilizing website information for decision-making, consumers exhibit distinct information requirements for these two goods categories. This underscores the pivotal moderating role of product type between external cues and online merchandise sales.

In summary, current research offers insights into the influence of external cues on consumers’ online purchase behavior. However, there remains a palpable gap, especially in harnessing objective data from e-commerce web pages. This study seeks to bridge this divide. While prior studies have mostly revolved around specific metrics from online comments, this research broadens the scope, probing diverse informational elements present on web pages. By integrating cue utilization theory with the under-utilized Elaboration Likelihood Model, this study offers a fresh lens to understand e-commerce consumer behavior, especially in the rich and complex environment of cross-border platforms.

## Research hypothesis and model

### Central path

#### Imported goods information quality

Within the framework of the Elaboration Likelihood Model, the central path of paramount importance is information quality. This stems from the fact that consumers inherently invest cognitive effort and thoughtful consideration when evaluating information quality. Existing scholarship has presented a multifaceted view of information quality assessment. Barnes & Vidgen have emphasized dimensions like website accuracy, timeliness, and the capacity to deliver trustworthy information [29]. DeLone & McLean have highlighted the role of information quality in reflecting the content aspect of e-commerce websites, encompassing content richness, relevance, and ease of comprehension [30]. Yang has contributed the notion that e-commerce website information quality pertains to the utility and adequacy of the content [31].

This study takes a nuanced approach, focusing specifically on the information quality associated with imported goods showcased on cross-border e-commerce platforms. For consumers engaged in purchasing imported goods, elevated information quality concerning these products has the potential to mitigate the perceived risk of compromised product quality. Consequently, this reduction in perceived risk significantly contributes to cultivating positive purchasing behavior among consumers.

To operationalize this central path, the study scrutinizes imported goods’ information quality, assessing it through the richness, ease of comprehension, and usefulness. This multifaceted evaluation hinges on three key dimensions: the length of product titles, the number of picture comments, and the count of additional comments. These dimensions align closely with the unique characteristics of the research platform, cross-border e-commerce. By investigating these dimensions, the study strives to provide a comprehensive understanding of how different external cues pertaining to imported goods can effectively ameliorate consumers’ perceived risk. Ultimately, this serves to reduce the uncertainty typically associated with online purchases, facilitating a more confident and decisive consumer approach towards online shopping.

*(1) Product title length*. This study focuses on the impact of product title length on consumer behavior in the context of imported goods in cross-border e-commerce. Longer product title length contain more keywords and provide a wider range of product details. Research by Hong Weiyin suggests that online display of product information facilitates customers in understanding the product and finding the desired item more quickly [32]. Kang’s findings indicate that more detailed product information leads to a better shopping experience by effectively informing customers [33]. Given that Chinese domestic consumers often lack knowledge about imported goods due to language barriers, relying on product title length becomes crucial in understanding product information and inferring product quality. In this study, the length of the product title on the web page is used to calculate the influence of product title length on sales. When the title contains more characters, it suggests that the product name is more rich and hence has a bigger impact on sales.

**H1a.** *Product title length positively affects imported products online sales*.

*(2) Picture comments*. This study investigates the influence of picture comments on consumer purchasing behavior in the context of imported goods on e-commerce platforms. Online comments serve as important reference signals for consumers, and the inclusion of pictures in these comments enhances the ability to convey product features and characteristics. Researchers have found that a higher quantity of picture comments has a positive impact on consumers’ purchase decisions [34]. Based on these findings, it can be inferred that an increased number of picture comments provides more detailed information for consumers, leading to a better understanding of the goods and ultimately influencing their purchasing behavior. Therefore, the assumption made in this study is that a higher number of picture comments will positively impact consumer purchasing behavior for imported goods.

**H1b.** *Picture comments positively affect imported products online sales*.

*(3) Additional comments*. This study examines the impact of additional comments on consumer purchasing behavior for products on e-commerce platforms. Initial comments are often based on limited knowledge of the product, whereas additional comments reflect users’ experiences and perspectives after using the product for a longer period. Empirical investigations have shown that the emotional content of additional comments has a stronger influence on product sales than initial comments [35]. Moreover, the quantity of additional comments provides consumers with more information and a perceived sense of usefulness for the product [36]. Additionally, buyers are more likely to provide honest feedback in their additional evaluations due to the smaller quantity compared to original comments [37]. Considering these findings, this study suggests that additional comments play a crucial role in shaping consumers’ perceptions of product quality and experience, and an increasing number of additional comments can impact consumers’ purchasing behavior positively.

**H1c.** *Additional comments positively affect imported products online sales*.

### Peripheral path

#### Source credibility

Source credibility plays a crucial role in consumers’ evaluation of online information. Research has shown that consumers perceive source credibility as an indicator of information reliability and trustworthiness [38]. In the context of cross-border e-commerce, where product legitimacy and quality may vary, the reputation of stores becomes a significant factor in assessing source credibility. Consumers tend to prefer stores with higher reputations to minimize the psychological risk of purchasing low-quality goods. Platforms like Tmall Global classify stores into brand flagship stores, official import retail stores, and brand franchises. Brand flagship stores and official import retail stores, which have official authorization from both the brand and the platform, are considered more credible sources. On the other hand, brand franchises operated by individuals may lack regulation, leading to concerns about the authenticity and quality of the imported products they sell, resulting in lower source credibility. In conclusion, in the context of cross-border e-commerce, the reputation of stores serves as an important cue for consumers to evaluate the credibility of sources. Stores with higher reputations are more likely to gain consumers’ trust and reduce their psychological risk, so that influence their purchasing behavior.

**H2.** *Source credibility positively affects imported products online sales*.

#### Collections

In the realm of cross-border e-commerce, the quantity of product collections serves as a pivotal indicator of product popularity and sales success, thereby influencing consumer purchase choices. Research has demonstrated that a higher number of collections correlates with increased sales, signaling greater recognition and acceptance by others. Consequently, this reduction in perceived choice risk enhances consumers’ likelihood to purchase the product [39, 40]. Given consumers’ potential limited knowledge of imported goods, they can employ the number of product collections as a gauge of popularity and quality. The larger the collection count, the more appealing the product becomes. This aligns with the peripheral path of the ELM model, enabling consumers to make judgments solely based on collection numbers. Hence, this study underscores product collections as a significant peripheral path influencing consumers’ online purchase decisions.

**H3.** *Collections positively affect imported products online sales*.

#### Price discount

Price discounts wield a substantial impact both physiologically and psychologically, motivating customers to amplify their purchases. As underscored by Kotler, price discounts foster favorable consumer behavior, culminating in heightened sales performance [41]. Similarly, Xiao’s research underscores the pivotal role of price discounts in shaping consumers’ perceived value, thus positively steering their purchase selections [42].

In the realm of cross-border e-commerce, where imported goods often carry premium price tags, consumers frequently assess product discounts prior to swift decisions. A discounted price eases the consumer’s purchase expenditure, consequently reducing their perceived financial risk. This cognitive process aligns with affective influences, resonating with the peripheral route of the Elaboration Likelihood Model. Consequently, this study asserts that price discounts wield a noteworthy impact on consumers’ online purchasing determinations within the cross-border e-commerce domain.

**H4.** *Price discount positively affect imported products online sales*.

#### Moderating effect of product-type

Research suggests that consumers have varying preferences when it comes to different types of goods. One common classification is based on whether consumers can access information about the product’s quality before making a purchase, dividing them into experience-based and search-based goods [25]. Experience-based goods are those that consumers can only evaluate after purchasing, while search-based goods are those for which consumers can gather quality information through relevant parameters prior to purchase. In daily life, electronic appliances such as refrigerators, cameras, and headphones fall into the category of search-based goods. Consumers can gain a general understanding of the quality of these goods by examining the parameters provided on the product web page. Conversely, skin care products and toiletries are considered experience-based goods. Each consumer’s perception of the same product can be subjective, and the quality can only be assessed through personal experience after the purchase. Building upon prior research, this study categorizes the products extracted from the Tmall Global website into two distinct types: search-based and experience-based. This classification aims to explore the divergent functions fulfilled by these product types within the framework of the association between external cues and product sales.

#### The moderating effect of product type between product title length and sales

Yanhui Zhang et al.’s research revealed that experience-based products place greater reliance on the length of review text compared to search-based products due to the limited availability of product information [43]. This is congruent with the findings of Ghose et al., who noted that longer review texts tend to encompass more product information and captivate consumer interest [44]. In the context of cross-border imported e-commerce, consumers often face a dearth of information about goods, compounded by the constraints of succinct product descriptions provided by websites. Consequently, when purchasing experience-based goods, consumers rely more on the goods’ titles to acquire meaningful information, which serves to enrich their understanding of the products, in contrast to the relatively well-informed purchase decisions made for search-based goods.

**H5a.** *The number of words in the product title length has a greater effect on product online sales for experience-based products compared to search-based products*.

#### The moderating effect of product type between picture comments and sales

An increase in the quantity of picture comments correlates with a heightened presentation of product information, enabling the portrayal of finer product intricacies. In the context of search-based products, consumers often appraise product quality through tangible parameters, rendering the demand for a substantial number of picture comments less pronounced. Conversely, with experience-based products, picture comments play a dual role. Not only do they provide consumers with a tangible glimpse of the product’s attributes, but they also serve as a means for gauging the extent of fellow consumers’ endorsement, thereby amplifying their impact on purchasing decisions.

**H5b.** *The number of picture comments has a greater effect on product online sales for experience-based products than for search-based products*.

#### The moderating effect of product type between additional comments and sales

Drawing from the preceding analysis, a similar line of reasoning indicates that, particularly with experiential goods, consumers face a shortfall in acquiring genuine product experience prior to making a purchase. In this context, additional comments serve as a form of compensation, attenuating the deficiency in firsthand consumer experience. Consequently, a higher count of additional comments engenders a more comprehensive portrayal of the product’s actual attributes. This compensatory effect mitigates the limitations arising from consumers’ limited grasp of product information before committing to a purchase, thereby elevating the likelihood of a purchase being made.

**H5c.** *The number of additional comments has a greater effect on product online sales for experience-based products than for search-based products*.*、*

#### The moderating effect of product type between source credibility and sales

In this study, the notion of information source credibility pertains to the status of stores operating within Tmall Global as either overseas flagship stores for the brand or directly managed by Tmall Global itself. These sources possess heightened credibility due to the endorsement and assurance provided by the official platform. Given the predominantly skincare and body care nature of the experience-based products investigated in this study, the stringent quality demands of these items, necessitated by their direct contact with the skin, are noteworthy. Furthermore, considering the susceptibility of imported goods in this category to counterfeiting, consumers exhibit a proclivity toward opting for sources with elevated credibility, thus favoring the purchase of goods from Tmall Global’s official flagship or directly managed stores.

Conversely, the search-based goods encompassed in this study predominantly comprise electronic and electrical items characterized by intricate production processes. In comparison to the former category, these goods are less susceptible to counterfeiting or imitation. As a result, the requisites for a flagship store’s endorsement are relatively less stringent in this context.

**H5d.** *Source credibility has a greater effect on product online sales for experience-based products than for search-based products*.

#### The moderating effect of product type between collections and sales

As indicated by the preceding analysis, the quantity of collected products serves as a readily discernible indicator that facilitates consumers in swiftly assessing the quality of a product. This indicator proves especially advantageous for experiential products, affording consumers a more convenient and efficient means to gauge product quality compared to search-based goods. Notably, the efficacy of this indicator is more pronounced in the case of experiential products, surpassing its utility for search-based products.

Furthermore, an increased count of favorites also holds significance for consumers of experiential goods, playing a vital role in shaping their emotional disposition towards the product. This augmented level of favorability provides substantial emotional support for consumers making purchases within the experiential product category.

**H5e.** *The number of collections has a greater effect on product online sales for experience-based products than for search-based products*.

#### The moderating effect of product type between price discount and sales

Mariola’s analysis posited that search-based goods exhibit superior monetary incentives within identical contexts [45]. Expanding upon this perspective, Kahn’s research observed that consumers, when acquiring search-based goods, are inclined to emphasize the price-to-performance ratio to optimize benefits [46]. In parallel, Bao Jinlong’s study discerned that for search-based goods characterized by diminished shopping risk, consumer attention tends to center on the product’s price during purchase deliberations [47]. Owing to the typically elevated prices associated with search-based goods, consumers channel heightened focus toward discount availability. Therefore when the goods have a high price discount, it will attract consumers of search-based goods to buy them.

**H5f.** *The number of price discount has a greater effect on product online sales for search-based products than for experience-based products*.

**Fig 1:**
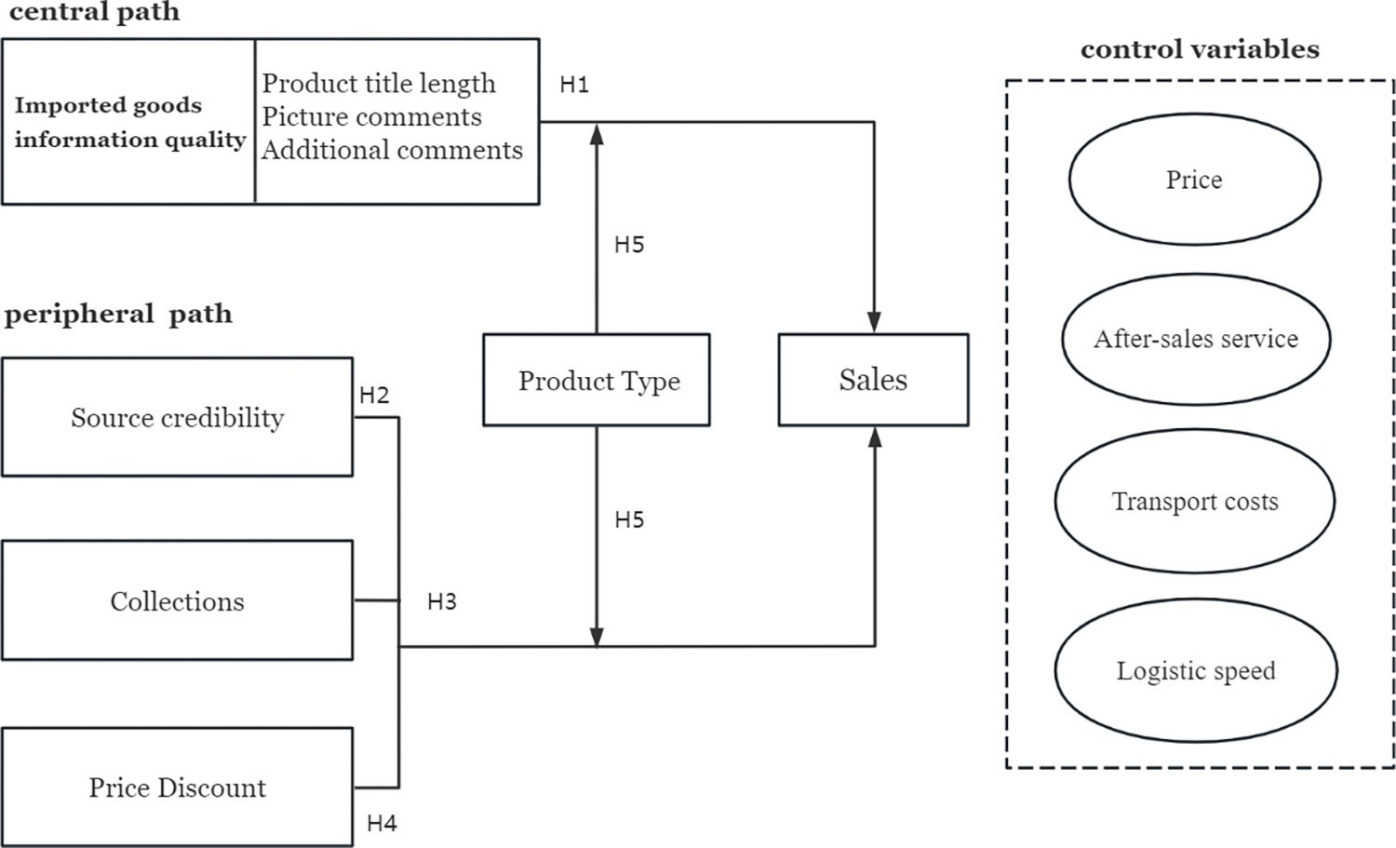
Conceptual framework.

In conclusion, this study develops a conceptual model of the variables affecting international e-commerce product sales (Fig 1).

## Materials and methods

### Data sources and preprocessing

This study investigates consumer behavior within the context of cross-border e-commerce platforms, focusing on the role of external cues in influencing product sales. The fundamental premise for consumers to generate demand for imported goods hinges upon the ability of cross-border e-commerce platforms to provide compelling and informative product details. In turn, consumers access and evaluate this information through external cues presented on the platform’s product pages. Consequently, our empirical investigation relies on data sourced from these digital touchpoints.

The study predominantly centers on the Tmall Global platform. Two primary rationales underscore the selection of this platform. Firstly, Tmall Global boasts the largest market share among Chinese cross-border e-commerce platforms dedicated to imports. According to data from the third-party market research firm eMarketer, as of the year 2022, Tmall Global commands a substantial global market share of 9.7%, positioning it as the second-largest player in the global cross-border e-commerce landscape, trailing only behind Amazon. Moreover, the financial report data from Alibaba Group for the year 2022 reveals that Tmall Global achieved an impressive Gross Merchandise Value (GMV) of 1.5 trillion RMB. This colossal GMV accounts for a substantial 39.6% of China’s cross-border import retail e-commerce market, firmly positioning Tmall Global as the market leader. Consequently, our choice to focus on Tmall Global ensures that our analytical findings possess wide-ranging applicability and relevance.

Secondly, the platform’s website design features facilitate the collection of data through web scraping, thereby minimizing the probability of data collection errors.

### Data collection

Data collection took place in August 2022. Initially, we employed the Octopus8.0 collection tool to gather information encompassing select product categories offered by Tmall Global. Given that skincare products and consumer electronics represent the primary product categories consumers tend to gravitate towards when engaging with cross-border e-commerce platforms, these categories were identified as the principal subjects of our study. The specific product categories included in our data collection effort are meticulously detailed in Table 1 below.

**Table 1:** Product collection type details.

Product Type	Subcategories	Product Details
Experience-based products	Facial Care	Face Creams
		Sunscreen Creams
		Essences
		Lotions
	Cleansing Products	Hair Shampoos
		Shower Gels
		Facial Cleansers
Search-based products	Large Home Appliances	Floor Cleaners
		Vacuum Cleaners
	Small Home Appliances	Food Processors
		Electric Kettles
		Electric Toothbrushes
	Digital Products	Smartwatches
		Bluetooth Earphones

**Fig 2:**
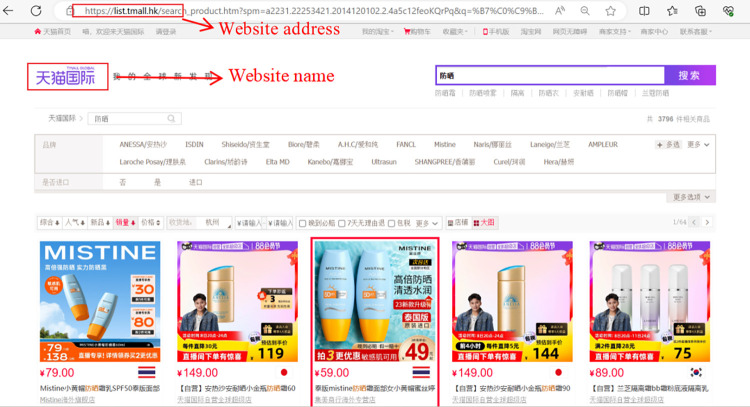
Example of Tmall Global website.

**Fig 3:**
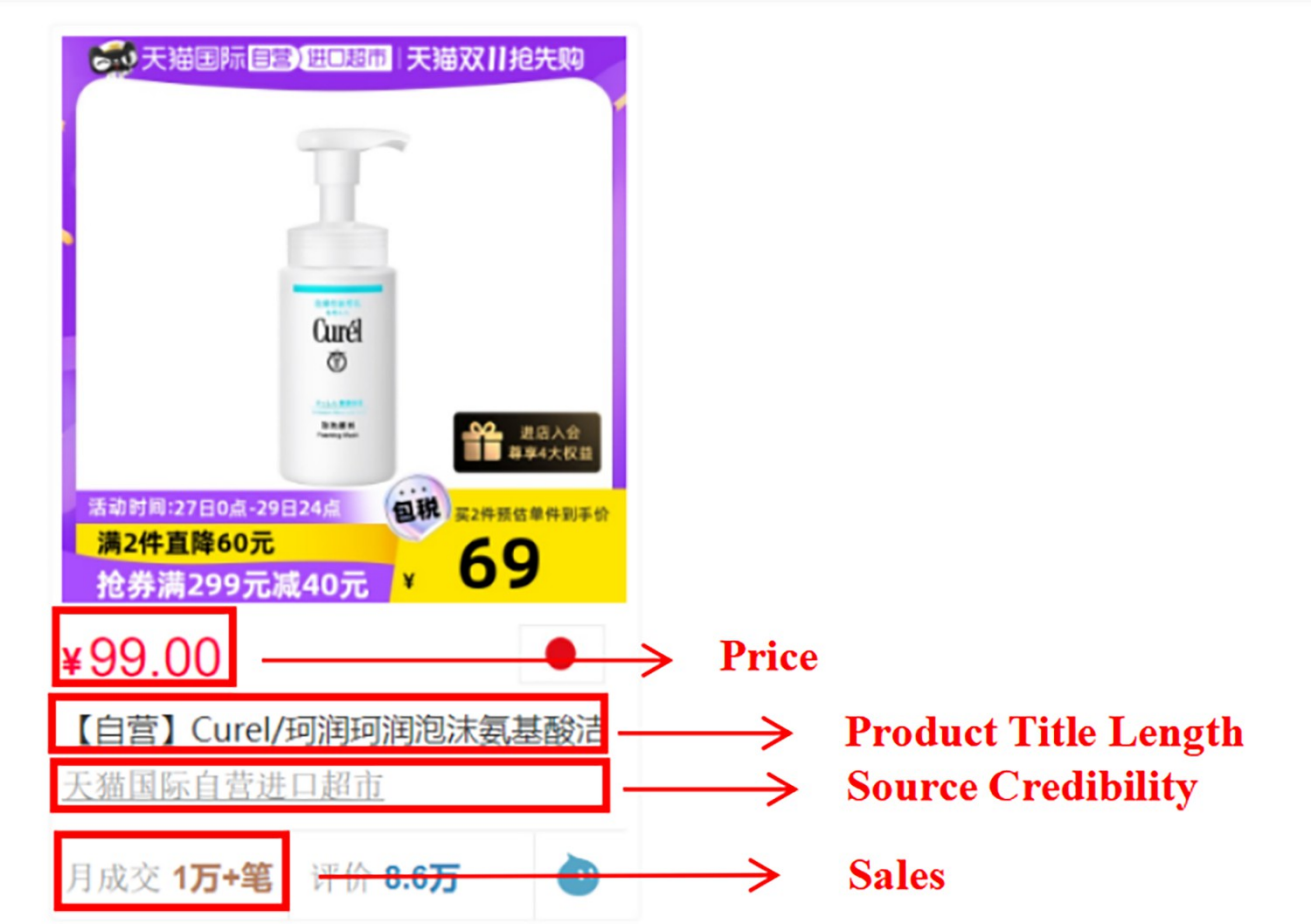
Example of Tmall Global product home page.

**Fig 4:**
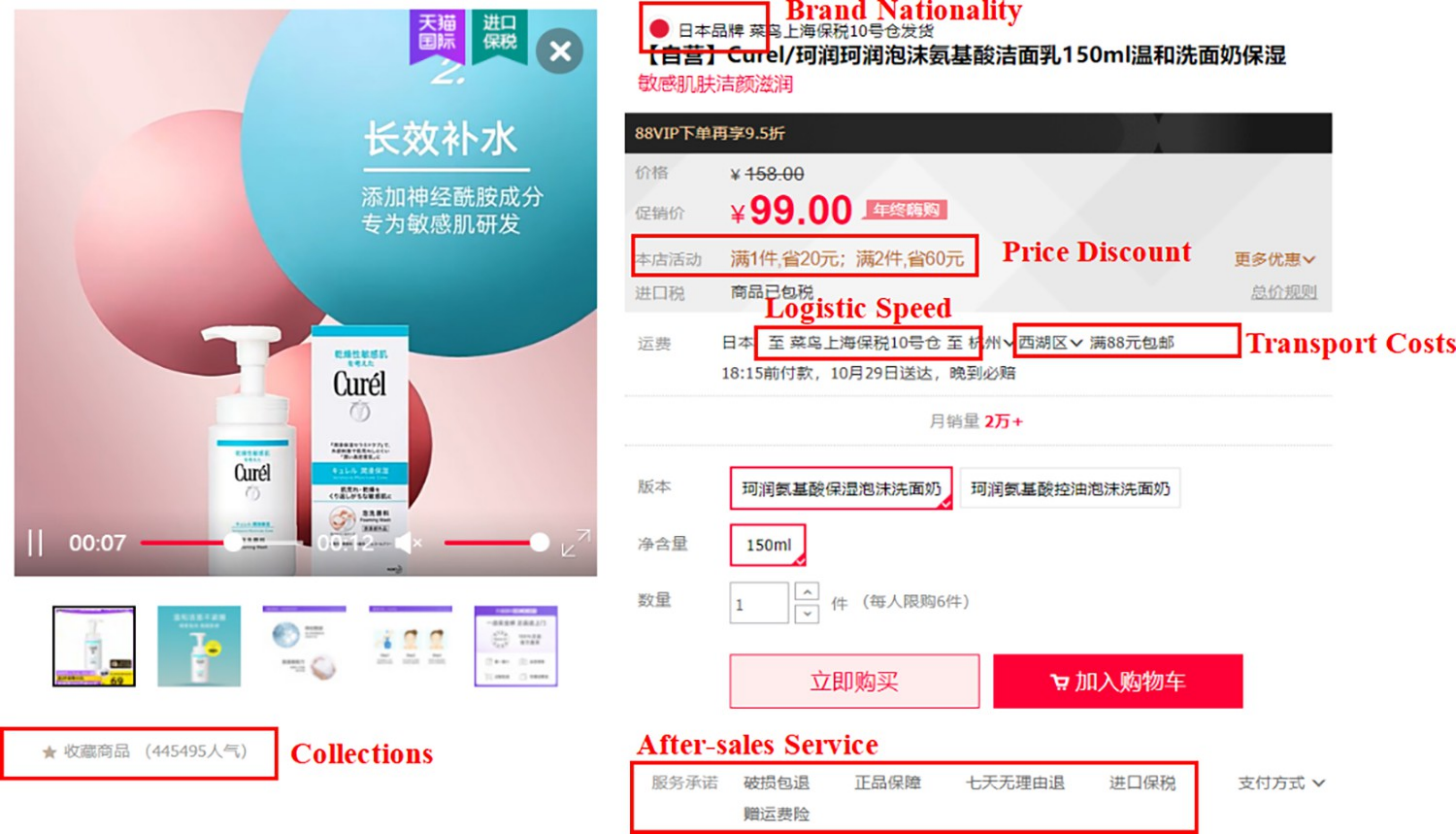
Example of Tmall Global product detail page.

**Fig 5:**
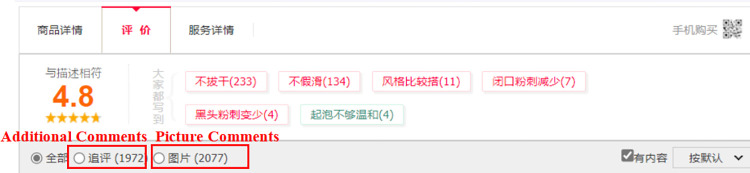
Example of Tmall Global product comments page.

Given the inherent diversity of product details, data collection efforts were bifurcated to encompass information available on product homepages as well as detailed product pages. An example of website and product pages (Figs 2–5)are shown below.

Detailed information was gathered for each product based on its specifications, including monthly sales, product title length, prices, store types, collections, brand nationalities, price discount, shipping costs, shipping locations, after-sales service, number of additional comments, and number of picture comments. The data collection process was efficiently completed within three hours to ensure data comparability. Subsequently, the collected data underwent thorough sorting and cleaning to eliminate any inaccurate or extreme values. The resulting data comprised 42,840 data points, representing a total sample of 2,141 goods. Specifically, the sample included 1,032 electrical devices and 1,109 skin care products. Please refer to Table 2 for a detailed distribution of the samples.

**Table 2:** Sample distributions.

Type	Frequency	Valid Percent	Cumulative Percent
Products Type			
experience-based products	1109	51.8	100.0
search-based products	1032	48.2	48.2
Stores Type			
Tmall Global supermarket&Tmall Global direct stores	1777	83.0	100.0
others	364	17.0	17.0
Logistics Speed			
from Chinese bonded warehouse	1834	85.7	100.0
others	307	14.3	14.3
Price Discount			
price discount available	1351	63.1	100.0
N/A	790	36.9	36.9
After-sales Service			
after-sales service available	1594	74.5	100.0
N/A	547	25.5	25.5
Transport Costs			
free shipment	1751	81.8	100.0
N/A	390	18.2	18.2

### Model

In the present study, we primarily focus on product sales as the dependent variable to elucidate consumer purchasing behaviors concerning imported goods. This is predominantly manifested through the augmentation of product sales demonstrated on product pages. Thus, the monthly sales volume of products depicted on these pages is employed as a proxy for the volume of product transactions.

Our explanatory variables are categorized into four principal elements impacting product sales volume: the quality of imported product information, source credibility, collections, and price discount. The quality of imported product information is characterized by the richness, understandability, and utility of information on import products offered by cross-border e-commerce platforms. Information richness is quantified through the character length of product titles, understandability through the picture comment count, and utility through the additional comment count, with data derived via web scraping.

Source credibility is evaluated based on the reliability and trustworthiness of the product source, utilizing store type as an indicator. Stores are classified into "Tmall Global import supermarket," "brand overseas flagship store," and other categories, with the first two denoting higher source credibility (assigned a value of "1") and the latter indicating lower credibility (assigned a value of "0").

Additionally, data regarding product collection count and price discount were amassed. To mitigate the possible influence of other product attributes on sales, control variables such as product price, after-sales service, transport cost, and logistics speed were incorporated. Product type serves as a moderating variable and is bifurcated into experience-based product (assigned a value of "1") and search-based product (assigned a value of "0").

To compensate for potential disparities in monthly transaction volumes amongst products—which can range from thousands to a minimal few—a logarithmic transformation was applied to all continuous variables. Crucially, the variables in our study were derived from objective data sourced from Tmall Global. A detailed presentation of the specific variables utilized, along with their corresponding measures, is provided in Table 3.

**Table 3:** Model variables and measures.

Variable Type		Variables	Variable Description
Dependent Variable		Sales	Number of monthly sales of products displayed on the page
Independent Variables	Central Path	Product Title Length	The length of the product title
		Picture Comments	Number of consumers comments containing pictures
		Additional Comments	Number of additional comments made by consumers to the initial review
	Peripheral Path	Source Credibility	Type of store the product belongs to (1 = Tmall import supermarket or brand overseas flagship store; 0 = others)
		Collections	The number of product collections displayed on the page
		Price Discount	Whether the page shows the price discount of the product (1 = Yes,0 = No)
Control Variables		Price	product’s ultimate selling price as stated on the product information page
		After-sales Service	Whether the product’s service guarantee states "seven days no reason to return" or "worry-free after-sale" (1 = Yes,0 = No)
		Transport Costs	whether the goods is shipped with postage as stated on the product details page. (1 = Yes,0 = No)
		Logistic Speed	whether a domestic Cainiao bonded warehouse is the shipment location indicated for the product (1 = Yes,0 = No)
Moderator Variable		Product Type	Type to which the product belongs (1 = experience-based product; 0 = search-based product)

### Descriptive statistics

Table 4 presents the descriptive statistics for the key variables, revealing important insights into the characteristics of the studied variables. The findings highlight a wide range of variation and a high degree of dispersion in the indicators of sales, number of picture comments and additional comments, price, and number of collections. These results underscore the significant disparities in business competence observed among different retailers.

**Table 4:** Descriptive statistics.

Variable Type		Variables	Obs.	Median	Std. Dev.	Min	Max
Dependent Variable		Sales	2,141	324.2	1,188	1	30,000
Independent Variables	Central Path	Product Title Length	2,141	34.07	3.690	18	67
		Picture Comments	2,141	158.5	498.8	1	16,192
		Additional Comments	2,141	66.41	224.2	1	7,154
	Peripheral Path	Source Credibility	2,141	0.830	0.376	0	1
		Collections	2,141	10,779	36,351	6	739,004
		Price Discount	2,141	0.631	0.483	0	1
Moderator Variables		Product Type	2,141	0.518	0.500	0	1
Control Variable		Price	2,141	877.9	2,214	8	30,110
		After-sales Service	2,141	0.745	0.436	0	1
		Transport Costs	2,141	0.818	0.386	0	1
		Logistic Speed	2,141	0.857	0.351	0	1

## Results

### Hypotheses testing

The econometric model employed in this study utilized ordinary least squares (OLS) for baseline regression analysis. The analysis was performed using Stata software. A hierarchical regression framework was employed to consider the effects of various variables. Initially, the central path variables and marginal path variables were included separately in the model, followed by the inclusion of all explanatory variables. Model 1 presents the regression outcomes featuring solely the control variables. Among these controls, the coefficient for Price stands at -0.448, signifying statistical significance at the 1% level. This suggests an inverse relationship, indicating that elevated goods prices are associated with diminished monthly sales. Meanwhile, the coefficient for logistics speed is observed at 0.640, also exhibiting significant positive correlation at the 1% threshold. This implies that enhanced logistics speed contributes to facilitated online sales augmentation. Additionally, it is worth noting that the control variables encompassing after-sales service and transportation cost do not manifest statistical significance. The same conclusion can be drawn from the full-sample regression results of the model 4.

Model 2 and Model 3 were used as baseline models to evaluate the significance of the central path and peripheral path on product sales. The degree of model fit was assessed by comparing the increase in R2. Notably, Model 3 exhibited a substantial improvement in fit, with an R2 value of 0.401, achieved through the incorporation of the complete range of central path and peripheral path variables.

#### The impact of information quality of imported goods on sales

The impact of the product title length on sales is found to be insignificant. In the baseline regression, the coefficient of the product name variable is positive (0.349), but it is not statistically significant at the conventional confidence levels of 1%, 5%, and 10%. This suggests that the length of the product title has a weak effect on the online sales of cross-border e-commerce products, thereby failing to support hypothesis H1a. It is speculated that longer titles may provide more information about the products, facilitating consumers’ understanding to some extent. However, titles with excessive content may overwhelm consumers and impede their reading experience, thereby discouraging purchase behavior.

The baseline regression results confirm that the number of picture comments has a significant positive impact on the online sales of cross-border e-commerce products. The coefficient of the number of picture comments variable is positive (0.152) and statistically significant at a level of p<0.001. This finding provides support for hypothesis H1b, indicating that an increase in the number of picture reviews is associated with higher online sales of the products.

The baseline regression results indicate that the number of additional comments does not have a significant effect on product sales, as the coefficient (0.036) is not statistically significant at the 1%, 5%, and 10% confidence intervals. This finding does not support hypothesis H1c, suggesting that the presence of additional comments does not significantly influence online sales of cross-border e-commerce products. It is speculated that the mixed nature of additional comments, which can include both positive and negative remarks, may lead to consumer reservations and impact their decision-making process. Consumers may become more cautious or uncertain about the product after reading a certain number of additional comments, potentially affecting their purchasing behavior.

#### The impact of source credibility on sales

The baseline regression results demonstrate a significant positive impact of the credibility of the information source on product sales, as indicated by the coefficient (0.414, p<0.001). This finding supports hypothesis H2, suggesting that consumers are more inclined to purchase imported goods from reputable sources such as Tmall Import Supermarket or the brand’s international flagship shop. The credibility of the store plays a crucial role in influencing consumer purchase decisions, as consumers are more likely to trust and rely on products from trusted and well-known sources.

#### The impact of collections of imported goods on sales

The results of the baseline regression (0.258, p<0.001) support hypothesis H3, which claims that a product’s sales increase with its collection. This result demonstrates how an important factor in a consumer’s decision to purchase a product is the amount of product collection.

#### The impact of price discount of imported goods on sales

The results of the baseline regression (0.305, p<0.001) demonstrate an extremely favorable impact of price discount on product sales. The empirical findings support hypothesis H4, which states that products are more likely to be purchased by customers when price discount are shown on their detail pages. All results are shown in Table 5.

**Table 5:** Baseline regression.

			DV = Sales			
Variable Type		Variables		Baseline Regression		
			**(1)**	**(2)**	**(3)**	**(4)**
Independent Variables	Central path	Product Title Length		0.742***		0.349
				(2.58)		(1.26)
		Picture Comments		0.191***		0.152***
				(4.54)		(3.78)
		Additional Comments		0.178***		0.036
				(4.06)		(0.84)
	Peripheral path	Source Credibility			-0.001	0.414***
					(-0.01)	(5.46)
		Collections			0.036**	0.258***
					(2.47)	(13.07)
		Price Discount			-0.491***	0.305***
					(-8.80)	(5.23)
Control Variables		Price	-0.448***	-0.398***	-0.449***	-0.450***
			(-17.84)	(-15.98)	(-19.94)	(15.41)
		After-sales Service	0.017	0.002	0.114*	0.072
			(0.24)	(0.03)	(1.76)	(1.12)
		Transport Costs	0.020	-0.241***	0.084	0.022
			(0.25)	(-3.10)	(1.13)	(0.30)
		Logistic Speed	0.640***	0.465***	0.350***	0.289***
			(6.76)	(5.30)	(4.07)	(3.38)
Adjusted R2			0.196	0.329	0.177	0.401
N			2141	2141	2141	2141
_Cons			6.433***	2.650***	7.415***	2.125**
			28.96	2.6	45.89	2.16

Note: Numbers in parentheses are standard errors.

*p < 0.1

**p < 0.05

***p < 0.01.

Robustness test

To ensure the robustness of the regression results, this study includes a second-period analysis using data from the same batch of Tmall Global products. The data for the second period were collected on August 10, 2022, and February 10, 2023, with a six-month gap between the two collections. The second-period data consisted of 2135 samples, accounting for potential changes in product availability over time. With the exception of additional comments, the findings from the second-period analysis showed no significant deviations from the baseline regression results. This further strengthens the reliability of the initial findings. The results are shown in Table 6.

**Table 6:** Robustness tests based on time samples.

Variable Type		Variables	Sales
Independent Variables	Central path	Product Title Length	0.136
			(0.57)
		Picture Comments	0.135***
			(3.59)
		Additional Comments	0.106**
			(2.44)
	Peripheral path	Source Credibility	0.508***
			(7.05)
		Collections	0.279***
			(13.72)
		Price Discount	0.398***
			(6.32)
Control Variables		Price	-0.353***
			(-14.19)
		After-sales Service	0.382***
			(5.49)
		Transport Costs	-0.337***
			(-4.71)
		Logistic Speed	0.043
			(0.40)
Adjusted R2			0.434
N			2135
_Cons			2.487***
			-2.89

Note: Numbers in parentheses are standard errors.

*p < 0.1

**p < 0.05

***p < 0.01.

Heterogeneity analysis of brand nationality

In consumer evaluations and brand judgments, consumers tend to be influenced by their perception of the overall country image. When consumers lack sufficient knowledge about other product attributes, they rely on the country of origin as a cue to make judgments. To examine the impact of brand nationality on product sales, this study categorizes the collected commodities into Asian countries and European and American countries based on brand nationality. Separate regressions are conducted to analyze the differences in the role of commodities from different brand affiliation countries on sales. The results in Table 7 indicate that commodities affiliated with European and American brands are more influenced by the number of picture comments, and store type plays a more significant role in influencing European and American commodities. Additionally, regardless of nationality, the number of collections and promotional activities are important factors influencing consumer purchasing decisions. However, the title of the product and the number of additional comments do not have a significant effect.

**Table 7:** Heterogeneity analysis.

DV = Sales				
Variable Type		Variables	(1)	(2)
			Asian countries	European and American countries
Independent variables	Central path	Product Title Length	0.277	0.462
			(0.58)	(1.36)
		Picture Comments	0.041	0.211***
			(0.62)	(4.14)
		Additional Comments	0.118	-0.026
			(1.64)	(-0.49)
	Peripheral path	Source Credibility	0.226*	0.598***
			(1.91)	(5.93)
		Collections	0.327***	0.219***
			(9.36)	(9.12)
		Price Discount	0.407***	0.255***
			(3.99)	(3.51)
Control variables		Price	-0.483***	-0.390***
			(-11.10)	(-12.01)
		After-sales Service	0.104	0.002
			(0.93)	(0.02)
		Transport Costs	-0.156	0.080
			(-1.27)	(0.69)
		Logistic Speed	0.209	0.334***
			(1.59)	(3.22)
Adjusted R2			0.416	0.384
N			828.000	1313.000
_Cons			2.683	1.542
			(1.56)	(1.29)

Note: Numbers in parentheses are standard errors.

*p < 0.1

**p < 0.05

***p < 0.01.

#### The moderating effect of product type

To assess the moderating effect of product type, this study included an interaction term between the moderating variable and the independent variable in the baseline model. Table 8 presents the regression results.

**Table 8:** Moderating effect of produce type.

DV = Sales							
	Model(1)	Model(2)	Model(3)	Model(4)	Model(5)	Model(6)	Model(7)
Product Title Length	0.060	0.312	0.300	0.309	0.435	0.312	0.404
	(0.17)	(1.12)	(1.08)	(1.10)	(1.58)	(1.12)	(1.10)
Picture Comments	0.161***	0.124***	0.152***	0.158***	0.190***	0.160***	0.365***
	(3.97)	(2.78)	(3.75)	(3.91)	(4.70)	(3.96)	(6.19)
Additional Comments	0.028	0.028	-0.031	0.032	0.002	0.029	0.094
	(0.65)	(0.66)	(-0.68)	(0.74)	(0.05)	(0.68)	(1.64)
Source Credibility	0.387***	0.417***	0.441***	0.376***	0.414***	0.393***	0.393***
	(5.02)	(5.41)	(5.71)	(2.91)	(5.46)	(5.13)	(2.98)
Collections	0.265***	0.267***	0.267***	0.263***	0.163***	0.263***	0.132***
	(13.22)	(13.30)	(13.35)	(13.15)	(6.36)	(13.18)	(4.40)
Price Discount	0.341***	0.333***	0.333***	0.340***	0.346***	0.408***	0.463***
	(5.52)	(5.39)	(5.41)	(5.47)	(5.67)	(4.92)	(5.46)
Product Type	-0.115	-0.113	-0.119	-0.158	-1.546***	-0.031	-1.729***
	(-1.52)	(-1.50)	(-1.59)	(-0.93)	(-6.38)	(-0.30)	(-5.09)
Product Title Length×Product Type	0.637						0.083
	(1.13)						(0.15)
Picture Comments×Product Type		0.061*					-0.325***
		(1.80)					(-3.98)
Additional Comments×Product Type			0.119***				0.265***
			(3.42)				(3.13)
Source Credibility×Product Type				0.036			0.015
				(0.22)			(0.09)
Collections×Product Type					0.189***		0.222***
					(6.16)		(5.60)
Price Discount×Product Type						-0.154	-0.213*
						(-1.24)	(-1.71)
Control Variables	YES	YES	YES	YES	YES	YES	YES
_Cons	3.293***	2.472**	2.563***	2.435**	2.570***	2.416**	2.716**
	(2.58)	(2.49)	(2.58)	(2.39)	(2.61)	(2.43)	(2.08)
N	2141	2141	2141	2141	2141	2141	2,141
Adjusted R2	0.398	0.398	0.401	0.398	0.408	0.398	0.416
F	118.869	119.144	120.315	118.698	123.978	118.907	89.09

Note: Numbers in parentheses are standard errors.

*p < 0.1

**p < 0.05

***p < 0.01.

Results revealed that incorporating the interaction term between product name and product type in model (1) yielded a coefficient of 0.637, but the moderating effect of product type was not significant. This could be attributed to the fact that consumers can gather certain product information from the title for imported goods, regardless of whether they are search-type or experience-type products. Thus, hypothesis H5a was not supported. On the other hand, both model (2) with the interaction term of the number of picture comments and product type (0.061, p<0.1) and model (3) with the interaction term of the number of additional comments and product type (0.119, p<0.001) exhibited significant positive coefficients, confirming the presence of a moderating effect. In essence, for experience-based products, the quantity of picture comments and additional comments had a greater impact on sales.

Model (4) combines source credibility and product type. The interaction term coefficient is 0.036, indicating that the moderating effect is not statistically significant. The type of store plays a crucial role in determining the reliability of the product’s source, and consumers aim to minimize online shopping risks regardless of the product type. Hypothesis H5d could not be confirmed.

In model (5), the regression coefficient for the interaction term between collections and product type is significantly positive (0.189, p<0.001). This suggests that collections have a stronger impact on sales for experience-based products compared to search-based products. Hypothesis H5e was confirmed.

Model (6) includes the interaction between price discount and product type. The regression coefficient is not significant (-0.154), indicating that there is little variation in how price discount affect sales for different product types. It is speculated that price discount are a crucial factor for Chinese consumers when making online purchases. They pay attention to price discount regardless of the product type, resulting in minimal impact of product type on their purchase behavior.

The results of the empirical regression highlight that consumers exhibit a heightened emphasis on certain factors when purchasing experiential goods. Notably, the number of additional comments and picture comments garners greater attention, signifying a conspicuous presence of the herd effect. This outcome aligns with the conclusions drawn by Li and Wu [28]. Plausible rationale for this observation could stem from Chinese consumers’ inclination to rely on others’ feedback while procuring foreign experiential products. The unfamiliarity with these goods and limited direct access could necessitate a heavier reliance on external opinions. Consequently, more extensive and informative picture comments and additional comments can significantly sway consumer purchase decisions.

Furthermore, the resonance consumers experience with others is more pronounced in the context of experiential goods, manifested by the significance of the number of collections. This attests to a stronger positive influence on consumers of such products. This could be attributed to the emotional resonance consumers establish with peers who exhibit similar preferences, thereby bolstering the perceived value and appeal of the experiential goods.

In summary, the empirical findings accentuate the nuanced purchasing behavior associated with experiential goods, underlining the role of peer influence, comprehensive comments, and emotional connection in driving consumer decisions within this category. All results are shown in Table 8. Moreover, the results of all hypothesis tests are shown in Table 9.

**Table 9:** Hypothesis conclusion.

	Hypothesis Path	Coefficient	T-value	P	Results
H1a	Product title length→Products online sales	0.349	-1.26	0.209	rejected
H1b	Picture comments→Products online sales	0.152	-3.78	***	supported
H1c	Additional comments→Products online sales	0.036	-0.84	0.404	rejected
H2	Source credibility→Products online sales	0.414	-5.46	***	supported
H3	Collections→Products online sales	0.258	-13.07	***	supported
H4	Price discount→Products online sales	0.305	-5.23	***	supported
H5a	Product title length * Product type → Products online sales	0.637	1.13	0.281	rejected
H5b	Picture comments * Product type → Products online sales	0.061	1.80	*	supported
H5c	Additional comments * Product type → Products online sales	0.119	3.42	***	supported
H5d	Source credibility * Product type → Products online sales	0.036	0.22	0.830	rejected
H5e	Collections * Product type →Products online sales	0.189	6.16	***	supported
H5f	Price Discount * Product type → Products online sales	-0.154	-1.24	0.229	rejected

Note

*p<0.1

**p<0.05

***p<0.001

#### The curvilinear effect of product title length on product sales in the central path

The study examined the relationship between product title length and sales, proposing an inverted U-shaped non-linear association. The baseline regression and robustness tests revealed that the impact of product title length on sales in the central path was not significant. Given that the link between trade names and sales is not always linear, this study seeks to investigate the non-linear relationship between the two in order to evaluate the relationship that exists between product title length and sales. Thus, by introducing the squared term of the product title length in the regression model, the results showed that both the main and secondary terms were significant. The coefficient of the squared term displayed a negative sign, indicating an inverted U-shaped relationship between the number of characters in product title length and its sales.

**Fig 6:**
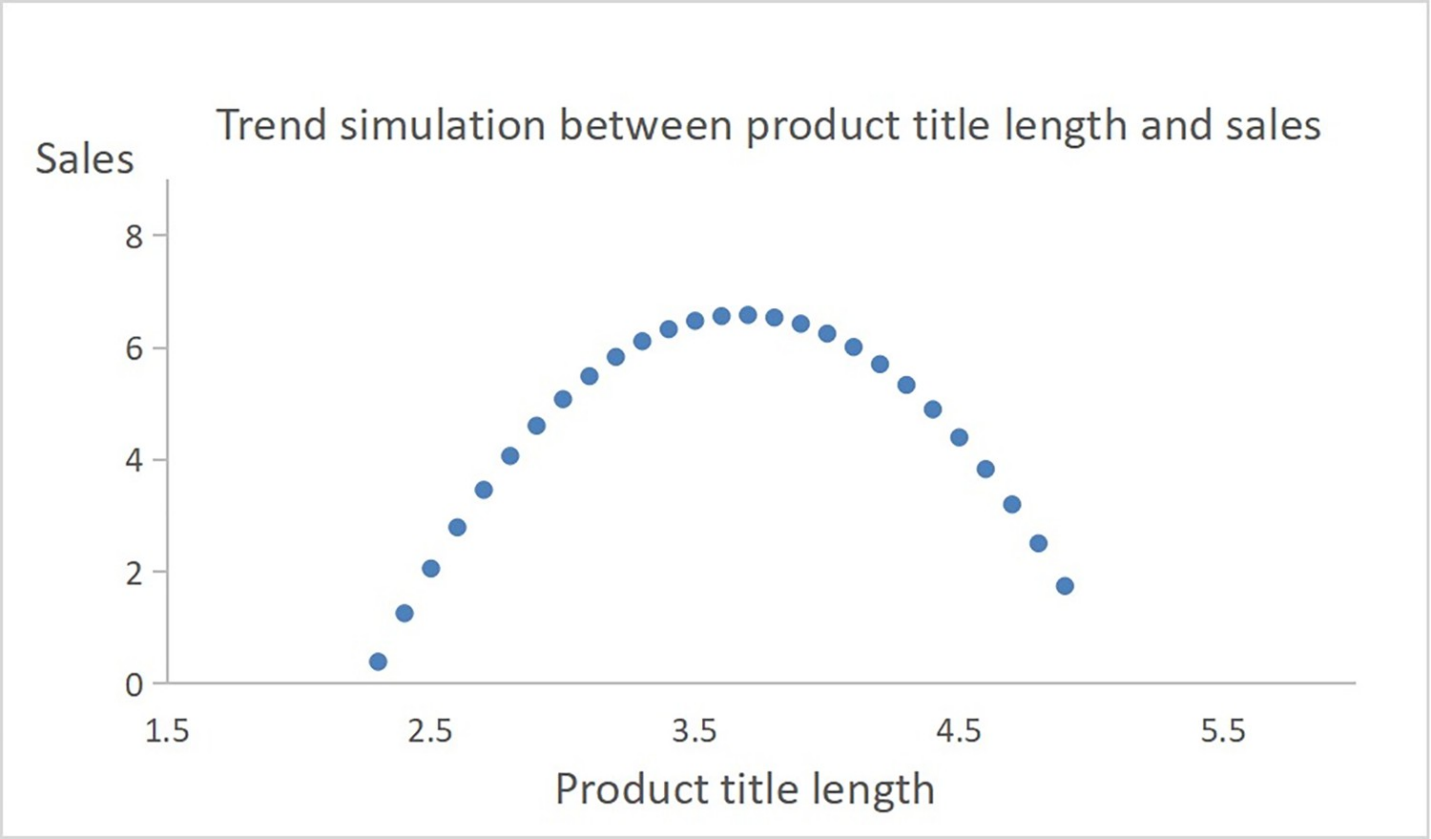
Trend simulation between product title length and sales.

To further validate this finding, a “utest” was conducted, confirming the existence of an inverted U-shaped connection in Table 10. A simulation using the regression coefficients demonstrated a clear inverted U trend between product title length and sales, providing additional support for the proposed theory (Fig 6). Hence, the study concluded that there is indeed a non-linear relationship resembling an inverted U between product title length and sales. Detailed results are shown in Table 11.

**Table 10:** Utest.

Utest	Lower bound	Upper bound
Interval	2.890	4.205
Slope	5.148	-3.388
P>|t|	0.001	0.026
Extreme point	3.683	
Overall P>|t|	0.0262	

**Table 11:** Inverted U relationship between product title length and sales.

Variable Type	Variables	Dependent variables
		Sales
Independent Variables	Product Title Length	23.918***
		(2.60)
	Product Title Length2	-3.247***
		(-2.51)
Control Variables	Price	-0.466***
		(-18.01)
	After-sales Service	-0.007
		(0.10)
	Transport Costs	0.039
		(-0.48)
	Logistic Speed	0.648***
		(-6.79)
Adjusted R2		0.282
N		2141
_Cons		-37.425**
		(-2.30)

Note: Numbers in parentheses are standard errors

*p < 0.1

**p < 0.05

***p < 0.01.

#### The moderating effect of product type on the relationship between product title length and sales

The study confirms the presence of an inverted U-shaped relationship between product title length and sales. The association varies across different types of products. For experience-based products, longer and redundant names limit information and decrease consumers’ desire to purchase, resulting in a steeper inverted U-shape. In contrast, for search-based products, where important information is conveyed in the name, longer names have minimal impact, leading to a flatter relationship.

**Fig 7:**
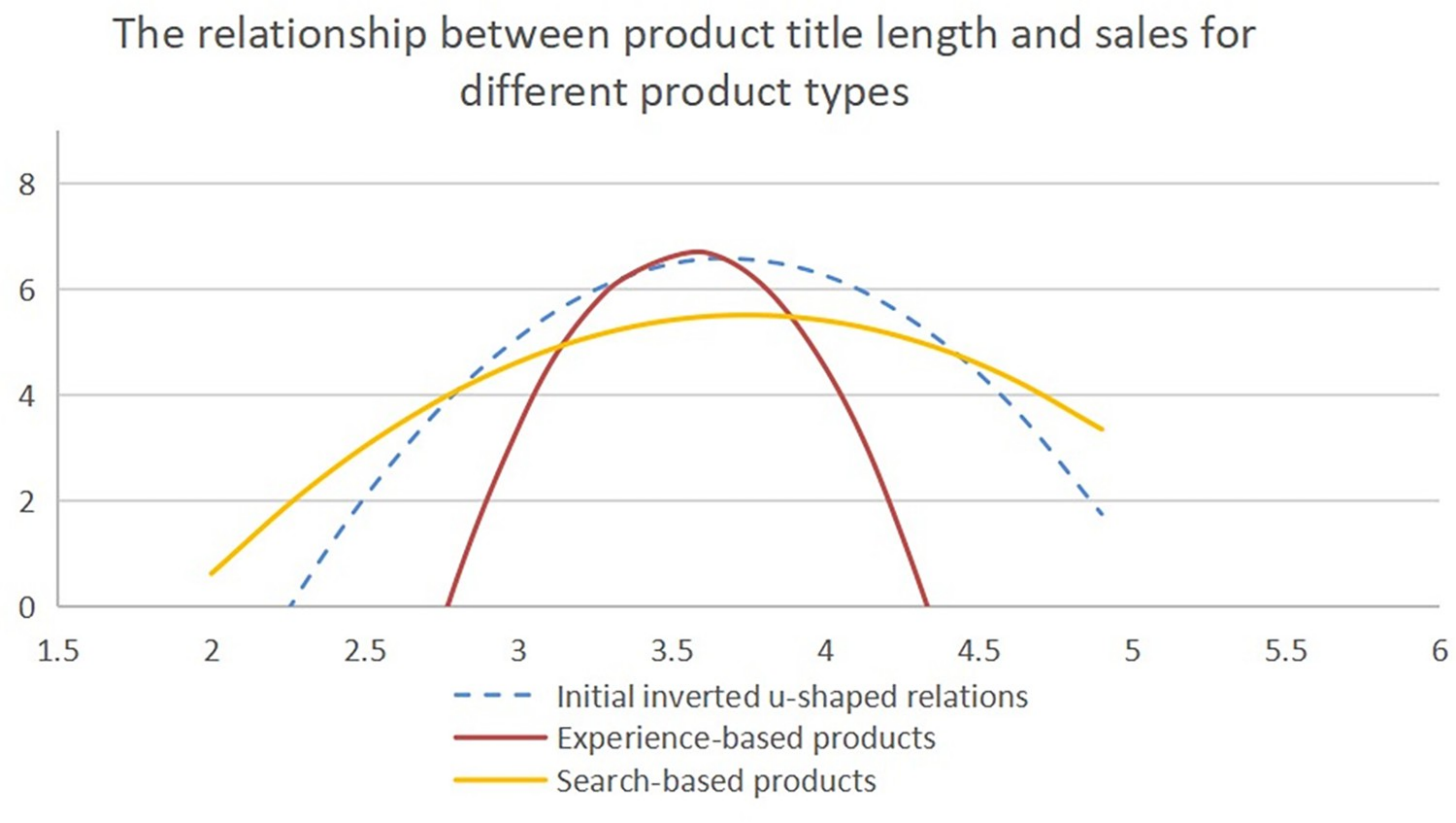
The relationship between product title length and sales for different product types.

The study incorporated the product name, squared term of the product name, product type, and their interaction terms into the regression. The analysis revealed a significant moderating effect when the coefficient of the interaction term between the secondary term of the product name and the product type was significant. The coefficient was significantly negative, indicating that as the number of characters in the name of an experiential product exceeds that of a search-based product, the inverted U-shaped curve becomes steeper. However, for search-based products, the curve flattens (Fig 7). The results are shown in Table 12. These findings provide insights into the optimal length of product title for different types of products. Marketers can tailor their naming strategies to maximize sales by considering the nonlinear nature of the relationship between product title length and consumer behavior. The study concludes by presenting a nonlinear adjustment trend graph that visualizes the relationship between product title length and sales, supporting the earlier hypothesis.

**Table 12:** Non-linear moderating effect of product type.

Variable Type	Variables	Dependent variables
		Sales
Independent Variables	Product Title Length	12.736
		(1.27)
	Product Title Length2	-1.677
		(-1.19)
Moderator Variable	Product Type	113.477**
		(2.05)
	Product Title Length×Product Type	63.878**
		(2.04)
	Product Title Length2×Product Type	-9.099**
		(-2.04)
Control Variables	Price	-0.358***
		(-11.53)
	After-sales Service	0.030
		(0.41)
	Transport Costs	-0.007
		(-0.08)
	Logistic Speed	0.637***
		(6.72)
Adjusted R2		0.378
N		2141
_Cons		-18.369
		(-1.02)

Note: Numbers in parentheses are standard errors.

*p < 0.1

**p < 0.05

***p < 0.01.

### Discussion

Picture comments significantly bolster sales of cross-border e-commerce products. These findings are consistent with prior research that highlights the positive association between picture comments and purchase intent [48, 49]. Within the cross-border e-commerce environment, consumers seem to value picture comments as a reliable indicator of product quality. Product source credibility exerts a strong positive effect on sales. Such observations are in line with Cha & Zhang [50] and Zhang & Jiang [51], emphasizing the pivotal role of source credibility. In scenarios typified by information asymmetry, such as cross-border purchases, consumers lean heavily on source credibility to gauge product quality.

The influence of additional comments on sales isn’t significant, diverging from the conclusions of Kim [36] and Shi [52]. This discrepancy may arise from the mixed nature of additional comments, comprising both positive and negative feedback. Such diversity may enhance consumers’ skepticism, impacting purchase decisions.

Both product collections, as indicated by crawling data, and price discounts markedly drive sales upwards. This is congruent with research findings by Liu [53] and Li [54], accentuating the role of popularity and price reductions in fostering purchasing intent.

Contrasting with Chen & Bowen’s [55] assertions, this study discerns an inverted U-shaped correlation between product title length and sales, presenting a nuanced perspective on the interplay between title length and sales. It offers insights into the intricate patterns underlying e-commerce consumer behaviors.

This study employs web crawlers for data extraction, diverging from conventional methodologies such as questionnaires. This innovation enhances the authenticity and impartiality of our findings. Moreover, the introduction of the ELM model into the e-commerce domain enables a nuanced categorization of external cues into central and peripheral pathways, refining consumer behavior analyses in e-commerce scenarios.

This investigation is bound by inherent limitations. Owing to the intricate nature of data extraction from cross-border e-commerce platforms and the limitations of web crawling tools, our data does not span multiple consecutive periods, precluding a thorough panel data scrutiny. The robust anti-crawling measures on some Chinese e-commerce websites limited the study’s scope. Future inquiries could harness advanced data extraction tools to collate data from a broader spectrum of platforms, facilitating a comprehensive assessment of determinants influencing product sales.

### Conclusions and recommendations

This paper investigates the impact of external cues on consumers’ purchase decisions in cross-border online shopping using the ELM model and cue utilization theory. The study analyzes Tmall Global online sales, applying the central path and peripheral path of the ELM model. The findings indicate that picture comments in the central path and store types, collections, and price discount in the peripheral path positively affect product sales. However, product title length and additional comments in the central path do not significantly impact sales.

Additionally, an interaction effect is observed between the central and peripheral paths and product type, with experience-based products showing a stronger influence of picture comments, additional comments, and product collections on sales. The study also identifies a non-linear relationship between product title length and sales, where consumers’ desire to purchase decreases when the product title length become too lengthy.

Furthermore, the product type moderates this relationship, with an inverted U-shaped pattern more evident for experience-based products and a smoother relationship for search-based products. This research contributes to the understanding of cross-border e-commerce consumer behavior and expands the application of non-linear conditioning in this domain.

The study offers four recommendations for decision-making in cross-border e-commerce. Firstly, platforms should prominently display picture comments to enhance consumer access, while merchants should incentivize customers to provide picture comments, increasing consumer attention. Secondly, for imported goods, attractive price discount should be offered to create a perception of favorable prices. Platforms should innovate marketing strategies to enhance platform appeal and foster consumer loyalty.

Thirdly, platform design should focus on improving the collection function, enabling consumers to easily screen and compare products. Merchants should prioritize enhancing store and product credibility, collaborating with platforms and brands to gain consumer trust. Quality and reputation, especially of big brands, should be considered to reduce consumer perceived risk and increase the likelihood of purchase. Lastly, when operating experience-based goods, merchants should capitalize on the promotional impact of positive comments. Optimizing customer follow-up and picture comments mechanisms, along with rewarding serious reviewers, can boost consumer motivation to post comments.

Moreover, in the process of formulating titles for products, it is imperative for merchants to accentuate the most compelling attributes of the goods. As evidenced by the findings of this study pertaining to product titles, an optimum impact is observed when the average character count of the title approximates 39.766. At this juncture, the title encapsulates an optimal level of information content, effectively stimulating consumer purchasing behavior. Consequently, given the confined space for information dissemination within the product title, merchants are advised to strategically prioritize the exhibition of the commodity’s distinctive strengths and attributes, thereby maximizing the title’s promotional potential.

## Supporting information

S1 Data (XLSX)
